# The associations of gestational weight gain and midpregnancy lipid levels with placental size and placental-to-birth weight ratio: findings from a chinese birth cohort study

**DOI:** 10.1186/s12884-023-05991-x

**Published:** 2023-10-11

**Authors:** Kangdi Zhang, Xiaomin Jia, Wenjie Yu, Xin Cheng, Yingqing Li, Xinqiang Wang, Jie Wang, Zhenhua Li, Yicheng Mao, Jiawen Zhao, Tao Li, Maolin Chen, Guopeng Gao, Chengyang Hu, Shuangqin Yan, Xiujun Zhang

**Affiliations:** 1 Department of Epidemiology and Biostatistics, School of Public Health, Anhui Medical University, 81 Meishan Road, Hefei, 230032 China; 2 Ma’anshan Maternal and Child Health Hospital, Ma’anshan, 243000 China; 3 Department of Clinical Laboratory, The First Affiliated Hospital of Anhui Medical University, Hefei, 230032 China; 4 Department of Gynecology and Obstetrics, Ma’anshan Maternal and Child Health Hospital, Ma’anshan, 243000 China; 5 Department of Child Health Care, Ma’anshan Maternal and Child Health Hospital, Ma’anshan, 243000 China; 6 Department of Humanistic Medicine, School of Humanistic Medicine, Anhui Medical University, 81 Meishan Road, Hefei, 230032 China; 7 Key Laboratory of Population Health Across Life Cycle (Anhui Medical University), Ministry of Education of the People’s Republic of China, 81 Meishan Road, Hefei, 230032 China

**Keywords:** Gestational weight gain, Serum lipid, Placental weight, Placental volume, Placental-to-birth weight ratio

## Abstract

**Background:**

The placenta serves as the sole maternal organ responsible for transmitting nutrients to the fetus, playing a crucial role in supporting standard fetal growth and development. To date, only a small number of studies have investigated the impact of maternal gestational weight gain and lipid concentrations on placental development. This study aimed to explore the influence of weight gain during pregnancy and lipid levels in the second trimester on placental weight, volume, and the placental weight ratio.

**Methods:**

This birth cohort study encompassed 1,358 mother-child pairs. Placental data for each participant was gathered immediately post-delivery, and the study incorporated data on gestational weight gain throughout pregnancy and lipid profiles from the mid-trimester. A linear regression model was employed to assess the correlations between gestational weight gain, mid-trimester lipid levels, and metrics such as placental weight, placental volume, and the placental-to-birth weight ratio (PFR).

**Results:**

In the study groups of pre-pregnancy underweight, normal weight, and overweight, the placental weight increased by 4.93 g (95% CI: 1.04–8.81), 2.52 g (95% CI: 1.04–3.99), and 3.30 g (95% CI: 0.38–6.22) per 1 kg of gestational weight gain, respectively. Within the pre-pregnancy underweight and normal weight groups, the placental volume increased by 6.79 cm^3 (95% CI: 3.43–10.15) and 2.85 cm^3 (95% CI: 1.31–4.39) per 1 kg of gestational weight gain, respectively. Additionally, placental weight exhibited a positive correlation with triglyceride (TG) levels (β = 9.81, 95% CI: 3.28–16.34) and a negative correlation with high-density lipoprotein (HDL-C) levels (β = − 46.30, 95% CI: − 69.49 to − 23.11). Placental volume also showed a positive association with TG levels (β = 14.54, 95% CI: 7.69–21.39). Conversely, PFR demonstrated a negative correlation with increasing HDL-C levels (β = − 0.89, 95% CI: − 1.50 to − 0.27).

**Conclusions:**

Gestational weight gain was significantly correlated with both placental weight and volume. This association was especially pronounced in women who, prior to pregnancy, were underweight or of normal weight. Additionally, TG and HDL-C levels during the mid-trimester were linked to placental development.

**Supplementary Information:**

The online version contains supplementary material available at 10.1186/s12884-023-05991-x.

## Introduction

Weight gain and metabolic shifts during pregnancy are typical physiological responses. Generally, a healthy woman gains between 8 and 12 kg during pregnancy, which is considered within the acceptable range [1]. A study conducted by Hu et al. involving 1,820 pregnant women in Tengzhou City, Shandong Province, China, indicated that gestational weight gain below the Institute of Medicine (IOM) standard was associated with an increased risk of low-birth-weight. Conversely, weight gain above the IOM standard heightened the risk of macrosomia. Appropriate weight gain during pregnancy is crucial for the healthy and safe development of the fetus. Pregnant women who gain insufficient weight, or less than ten pounds throughout the gestation period, are at a higher risk of delivering low-birth-weight babies and encountering preterm births [1, 2]. On the other hand, excessive weight gain, amounting to 40 to 50 pounds or more during pregnancy, predisposes the offspring to obesity, hyperglycemia, and hyperlipidemia [3]. Concurrently, all lipid components in a pregnant woman’s body may substantially increase, potentially doubling in certain instances. These physiological alterations are vital for the placenta and fetus, ensuring optimal intrauterine growth and development. Nonetheless, excessive weight gain, coupled with abnormal maternal lipid and lipoprotein metabolism, can significantly elevate the risk of complications such as gestational diabetes, macrosomia, pancreatitis, and preeclampsia. This not only impacts pregnancy outcomes but also augments the risk of future cardiovascular diseases in the newborn [4, 5].

The placenta serves as the sole direct link between the mother and fetus, acting as a vital conduit for nutrient transfer. As such, a robust and efficient placenta is indispensable for nurturing the fetus. Placental growth and development are typically gauged by its weight and volume. Studies have indicated that fetuses experiencing intrauterine growth restriction often exhibit a discernible reduction in placental weight and volume compared to those with standard birth weights [6]. There is an increasing consensus that evaluating the placenta is pivotal to understanding the underlying causes of intrauterine growth restriction. The placental-to-birth weight ratio (PFR) represents the ratio of the placental weight to the newborn’s birth weight. A general consensus posits that variations in PFR, whether increases or decreases, mirror the placenta’s operational efficiency and its nutrient transport capability. Thus, PFR is widely recognized as a marker of placental efficiency [7]. Past research has identified a U-shaped correlation between adverse perinatal outcomes, such as stillbirth and pre-eclampsia, and PFR [8–10]. Extremes in placental weight and volume, whether disproportionately large or small, can influence adverse pregnancy outcomes and cast a long shadow over the offspring’s predisposition to future diseases [7].

Wang et al. [11] conducted a study in Guangdong Province, revealing that maternal exposure to high temperatures during pregnancy could elevate the PFR and diminish placental weight and volume. Moreover, various factors associated with the gestational environment, such as induced hypoxia, alterations in circulating glucocorticoid and insulin-like growth factor levels, dietary constraints, and consistent maternal exercise, potentially influence placental weight and volume [12, 13]. The lipid metabolic status of mothers during mid-pregnancy has been linked to adverse birth outcomes, including small-for-gestational-age infants and macrosomia [14, 15]. Given the well-documented positive correlation between placental weight and neonatal birth weight [6, 16, 17], it stands to reason that lipid levels during mid-pregnancy could affect placental size and efficiency. In a recent study, Mitsuda et al. [18] posited a dose-response relationship between lipid concentrations and placental weight and efficiency. However, a comprehensive analysis of mid-pregnancy lipid levels was not presented.

It is postulated that early embryonic organ development is intricately linked to placental function. For instance, placental insufficiency might induce fetal cardiac insufficiency by narrowing coronary artery dimensions, subsequently elevating the risk of cardiovascular disease in adulthood [19, 20]. Thus, investigating the correlations between placental size variations, placental efficiency, pregnancy weight fluctuations, and mid-trimester lipid concentrations is crucial. Addressing these relationships not only plays a pivotal role in ensuring the fetus’s healthy birth but also forms a significant strategy in mitigating long-term adverse health outcomes for the newborn.

## Materials and methods

### Study design

Between April and July 2021, the project recruited eligible pregnant women from Ma’anshan Maternal and Child Health Hospital (MMCH). Data were collected through questionnaires at three stages: during early pregnancy at enrollment, during mid-pregnancy at the glucose tolerance test, and one week before delivery in late pregnancy. Ma’anshan City, located in eastern Anhui Province, comprises three districts (Bowang, Huashan, and Yushan) and three counties (Dangtu, Hexian, and Hanshan). The study sample was drawn from the MMCH, centrally situated in Ma’anshan City and affiliated with Anhui Medical University as the Ma’anshan Health Teaching Hospital. Pregnant women across all districts and counties who visit MMCH in their early pregnancy to establish a maternal health handbook and receive antenatal checkups meet the criteria for study population data collection. Approximately 60% of these women will be referred to the department of obstetrics at the MMCH, which records an average of 600 deliveries per month.

### Study participants

**Fig. 1:**
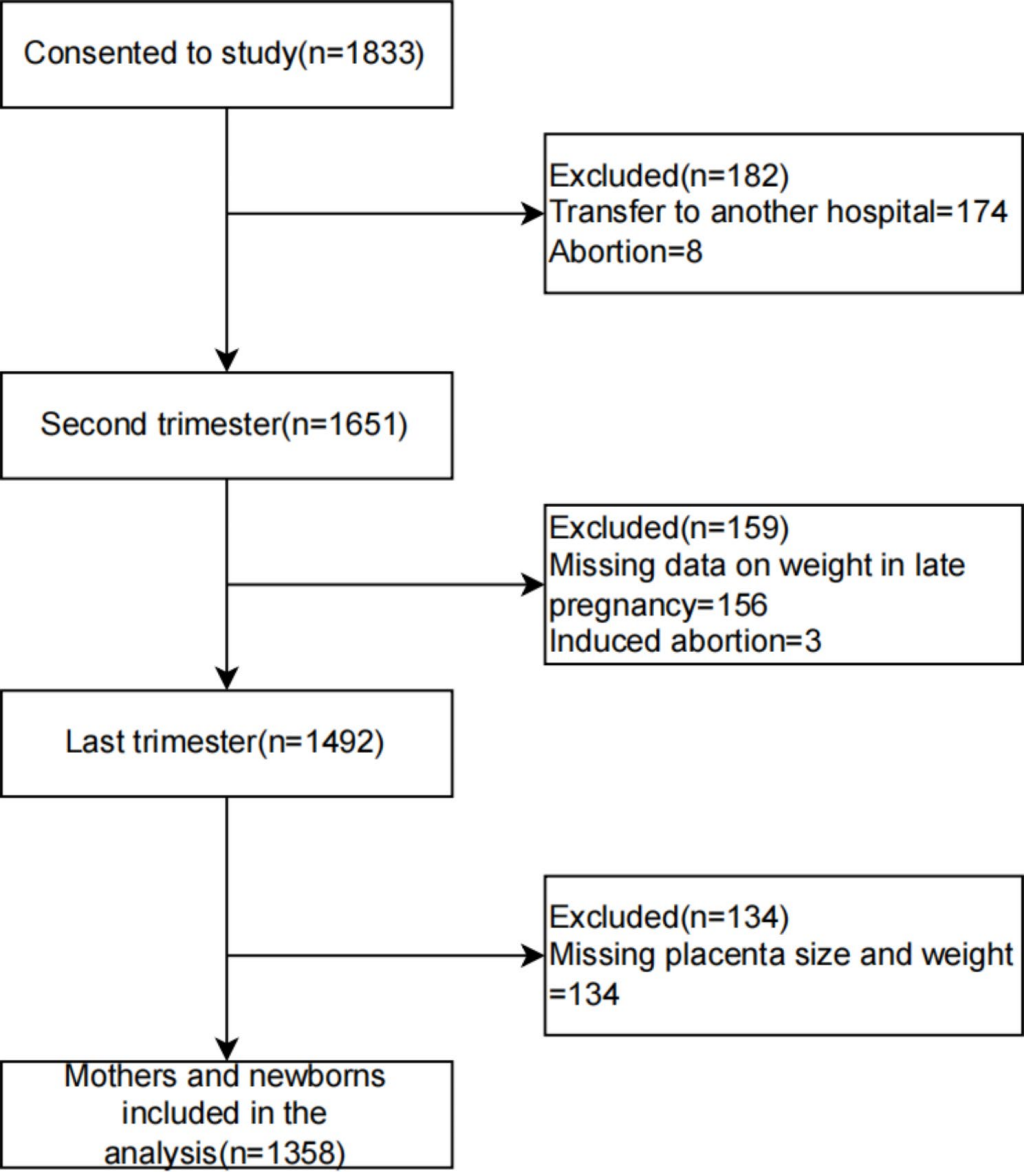
Flow chart of study participants included in the analysis

Pregnant women who underwent consistent maternity checkups at the hospital and could be followed until delivery were included in this study. The inclusion of study participants followed the following inclusion and exclusion criteria: (1) singleton, spontaneous conception; (2) gestational week ≤ 14 weeks at the time of registration [21]; (3) voluntary participation with the capability to complete the survey independently. For the exclusion criteria: (1) medical abortion, therapeutic induction of labor, or ectopic pregnancy; (2) pregnant women with infectious diseases, including hepatitis B, AIDS, syphilis, etc.; (3) those who were diagnosed with dyslipidemia-related diseases before pregnancy or who were taking lipid control drugs during pregnancy. Between April and July 2021, 1,833 pregnant women were initially enrolled. Of these: 174 were lost in the middle trimester because of transfers and 8 due to abortions; 156 were excluded in the late stage owing to absent weight data; 3 were lost due to induced labor; and 134 were excluded due to missing placenta size and weight. Thus, the study finalized with a cohort of 1,358 pregnant women (Fig. 1). Every participating woman provided written informed consent and the study received approval from the Ethics Committee of Anhui Medical University (No. 20,200,592).

### Data collection

A trained researcher guided pregnant women in completing the Maternal and Infant Health Assessment Registration Form within the Early Pregnancy Registry. This form captured demographic data, obstetric history, medical history, behavioral patterns during pregnancy, and the pre-pregnancy weight of the expectant mothers. Furthermore, a clinician measured current height, weight, blood pressure, and fetal heart rate. Pre-delivery weight was extracted by the researcher from participants’ perinatal handbooks, while lipid data from the second trimester was sourced from MMCH’s clinical testing system. Post-delivery, the umbilical cord was severed by a trained researcher (a female postgraduate student at Anhui Medical University, who was trained by obstetricians, gynecologists and nurses before being granted access to the maternity ward and operating room. No other medical activities were allowed except for operations related to placenta measurement and weighing). Excess blood was absorbed using gauze. The weight of placenta was then determined using an electronic scale (model: CN-LP20001) to a precision of 0.1 g. Subsequently, the fetal membranes were removed, the placenta was flattened, and its dimensions were measured. The longest diameter was determined, and its perpendicular counterpart was identified as the wide diameter. Thickness was gauged at the placenta’s thickest section. These measurements, accurate to 0.1 cm, were repeated thrice for an average value. A nurse weighed the newborn using a human electronic scale (model: DHM-3000) following cord severance and noted the gender of infant.

Pre-pregnancy BMI was calculated as: BMI = weight (kg) / [height (m)]^2^. Based on the Guidelines for the Prevention and Control of Overweight and Obesity in Chinese Adults [22], BMI was categorized: BMI < 18.5 kg/m^2^ as underweight; 18.5 kg/m^2^ ≤ BMI ≤ 23.9 kg/m^2^ as normal; 24 kg/m^2^ ≤ BMI ≤ 27.9 kg/m^2^ as overweight; and BMI > 27.9 kg/m^2^ as obese. Gestational weight gain was determined by subtracting pre-pregnancy weight from maternal weight before delivery [23]. Dyslipidemia diagnosis followed general adult criteria, where abnormalities were marked by specific thresholds for triglyceride, low-density lipoprotein, high-density lipoprotein, and cholesterol [24]. Placental volume and PFR were calculated based on referenced Eqs. [7, 25].

$$\eqalign{ placental\, volume=\frac{4}{3}\pi & \times \frac{placenta\, length\, diameter}{2} \cr & \times \frac{placenta\, width\, diameter}{2} \cr & \times \frac{placenta\, thickness}{2}}$$

$$PFR=\frac{placenta\, weight}{birth\, weight}\times 100$$

### Statistical analysis

Data from the questionnaire were inputted using Epidata 3.1 and analyzed with SPSS 23.0. Continuous variables that adhered to a normal distribution were presented as mean ± standard deviations (x̄ ± s). Comparisons between groups for these variables were performed using the t-test or ANOVA. Categorical variables were described as frequency (percentage) [N (%)]. For comparing dichotomous and unordered multicategorical variables between groups, the chi-square test was employed. In contrast, ordered multicategorical variables were compared using the rank sum test. Multiple linear regression assessed the effects of gestational weight gain and mid-trimester lipid levels on placental weight, placental volume, and PFR. The significance level was set at 0.05, and p-values less than 0.05 were deemed statistically significant.

## Results

### Baseline demographic characteristics and clinical information

Table 1 displays the baseline and clinical data of the pregnant women in this study, which includes a total of 1,358 mother-infant pairs. Of these, 714 (52.6%) were male offspring, and 644 (47.4%) were female. For all pregnant women, the average placental weight was (565.22 **±**106.60) g. Pregnant women with an education level of junior high school or below had a higher placental weight compared to other educational groups. Moreover, those who delivered before 37 weeks of gestation had a significantly lower placental weight (*p* < 0.05).

The mean placental volume across all pregnant women was (399.63 ± 111.18) cm^3^. Notably, smaller placental volumes were observed in pregnant women who delivered before 37 weeks of gestation and those with a pre-pregnancy BMI categorized as “wasting” (*p* < 0.05). The average PFR for all pregnant women was 17.09 (2.75). Women with a history of more than two pregnancies and only one delivery exhibited a lower PFR than all other groups. In contrast, a higher PFR was evident in women who delivered before 37 weeks of gestation compared to their counterparts (*p* < 0.05).

**Table 1:** Baseline demographic characteristics and clinical information of pregnant women

Variables	N (%), Mean ± standard deviation	Placenta weight(g)Mean ± standard deviation	t/F	P	Placenta volume (cm^3^) Mean ± standard deviation	t/F	P	PFR Mean ± standard deviation	t/F	P
Age (years)										
< 26	275 (20.2%)	561.77 ± 105.02	0.81^a^	0.491	392.28 ± 103.39	1.47^a^	0.220	17.11 ± 2.88	2.48^a^	0.060
26 ~ 30	668 (49.2%)	569.28 ± 105.76			403.41 ± 112.39			17.24 ± 2.74		
31 ~ 35	345 (25.4%)	562.61 ± 104.75			394.61 ± 111.75			16.88 ± 2.62		
> 35	70 (5.2%)	552.80 ± 128.28			417.14 ± 124.43			16.49 ± 2.94		
Education level										
Junior High School and below	246 (18.1%)	574.81 ± 112.21	4.77^a^	0.009	408.58 ± 110.81	1.89^a^	0.151	17.04 ± 2.76	0.80^a^	0.450
High school/junior college/college	622 (45.8%)	570.63 ± 111.51			401.64 ± 114.90			17.19 ± 2.93		
Bachelor’s degree or above	490 (36.1%)	553.53 ± 95.98			392.57 ± 106.24			16.98 ± 2.51		
Residence										
Rural	293 (21.6%)	560.50 ± 101.08	-0.86^b^	0.393	401.92 ± 106.69	0.40^b^	0.690	16.83 ± 2.74	-1.80^b^	0.073
Urban	1065 (78.4%)	566.52 ± 108.07			398.99 ± 112.42			17.16 ± 2.75		
Maternal Smoking										
Never	1303 (95.9%)	565.05 ± 106.46	0.71^a^	0.546	399.57 ± 111.84	0.06^a^	0.982	17.07 ± 2.71	1.56^a^	0.198
Now	1 (0.1%)	720.00			408.41			22.36		
Used to smoke, stopped after pregnancy	29 (2.1%)	564.61 ± 122.92			406.44 ± 101.55			17.15 ± 3.77		
Used to smoke and have quit for more than 3 months	25 (1.9%)	568.24 ± 94.74			394.42 ± 90.62			17.62 ± 3.30		
Exposure to secondhand smoke during pregnancy										
Yes	226 (16.6%)	566.03 ± 106.44	-0.13^b^	0.900	402.74 ± 110.65	-0.46^b^	0.644	17.21 ± 2.81	-0.76^b^	0.447
No	1132 (83.4%)	565.05 ± 106.67			399.00 ± 111.32			17.06 ± 2.74		
Alcohol consumption										
Never	1206 (88.8%)	565.81 ± 108.17	0.12^a^	0.948	400.35 ± 110.86	0.80^a^	0.494	17.08 ± 2.75	0.82^a^	0.480
Now	2 (0.1%)	570.00 ± 141.42			411.86 ± 83.97			17.48 ± 5.17		
Used to drink, stopped drinking after pregnancy	62 (4.6%)	561.21 ± 89.46			408.49 ± 123.59			16.75 ± 2.73		
Used to drink, and have quit for more than three months	88 (6.5%)	559.76 ± 96.43			383.16 ± 106.95			17.44 ± 2.81		
Gestational hypertension										
Yes	54 (4.0%)	570.60 ± 132.34	0.38^b^	0.705	428.33 ± 148.65	1.46^b^	0.150	17.24 ± 2.72	0.41^b^	0.680
No	1304 (96.0%)	564.99 ± 105.45			398.44 ± 109.26			17.08 ± 2.76		
Gestational diabetes										
Yes	246 (18.1%)	565.49 ± 116.67	0.04^b^	0.967	411.80 ± 108.17	1.90^b^	0.058	16.90 ± 2.78	-1.15^b^	0.249
No	1112 (81.9%)	565.16 ± 104.29			396.93 ± 111.70			17.13 ± 2.75		
Number of pregnancy										
1	605 (44.5%)	564.13 ± 104.48	0.31^a^	0.733	397.55 ± 114.99	0.91^a^	0.403	17.30 ± 2.65	4.39^a^	0.013
2	368 (27.1%)	568.92 ± 109.06			396.32 ± 108.33			17.07 ± 2.77		
> 2	385 (28.4%)	563.38 ± 107.69			406.04 ± 107.73			16.77 ± 2.87		
Number of deliveries										
0	857 (63.2%)	565.98 ± 108.03	0.13^a^	0.880	398.71 ± 114.12	1.32^a^	0.269	17.32 ± 2.77	9.04^a^	< 0.001
1	480 (35.3%)	563.55 ± 103.58			399.55 ± 106.10			16.66 ± 2.68		
> 1	21 (1.5%)	572.42 ± 120.07			438.53 ± 100.24			17.19 ± 2.87		
Gestational age (weeks)										
< 37	42 (3.1%)	460.40 ± 106.29	6.57^b^	< 0.001	325.91 ± 91.06	4.39^b^	< 0.001	18.60 ± 3.63	-2.76^b^	0.008
≥ 37	1316 (96.9%)	568.56 ± 104.94			401.98 ± 110.98			17.04 ± 2.71		
Infant sex										
Male	714 (52.6%)	570.60 ± 107.02	1.96^b^	0.050	402.17 ± 108.96	0.89^b^	0.374	17.07 ± 2.75	-0.17^b^	0.869
Female	644 (47.4%)	559.25 ± 105.89			396.80 ± 113.61			17.10 ± 2.76		
Pre-pregnancy BMI										
Wasting	147 (10.8%)	557.22 ± 99.67	2.47^a^	0.060	373.18 ± 90.96	5.74^a^	0.001	17.32 ± 2.79	1.58^a^	0.194
Normal	880 (64.8%)	561.62 ± 101.71			397.46 ± 105.21			17.15 ± 2.74		
Overweight	251 (18.5%)	575.92 ± 117.29			415.59 ± 126.69			16.80 ± 2.67		
Obese	80 (5.9%)	585.97 ± 130.85			421.91 ± 142.82			16.85 ± 3.06		
Gestational weight gain (kg)	13.99 ± 4.85	565.22 ± 106.60	-	-	399.63 ± 111.18	-	-	17.09 ± 2.75	-	-
Mid-pregnancy lipid profile										
TC (mmol/L)	5.78 ± 1.00	565.22 ± 106.60	-	-	399.63 ± 111.18	-	-	17.09 ± 2.75	-	-
TG (mmol/L)	2.46 ± 0.89	565.22 ± 106.60	-	-	399.63 ± 111.18	-	-	17.09 ± 2.75	-	-
HDL-C (mmol/L)	1.49 ± 0.24	565.22 ± 106.60	-	-	399.63 ± 111.18	-	-	17.09 ± 2.75	-	-
LDL-C (mmol/L)	3.16 ± 0.89	565.22 ± 106.60	-	-	399.63 ± 111.18	-	-	17.09 ± 2.75	-	-

^a^ The value is the F value; ^b^ the value is the t value.

Abbreviations: BMI, Body mass index; TC, cholesterol; TG, triglyceride; HDL-C, high-density lipoprotein; LDL-C, low-density lipoprotein; PFR, placental-to-birth weight ratio.

### Associations of gestational weight gain with placental weight, placental volume, and PFR

The one-way ANOVA results revealed that gestational weight gain values were (15.05 ± 4.37) kg, (14.43 ± 4.61) kg, (12.52 ± 5.22) kg, and (11.92 ± 5.48) kg for the pre-pregnancy underweight, normal, overweight, and obese groups, respectively. A statistically significant inverse relationship was observed between pre-pregnancy BMI and gestational weight gain (F = 17.93, *p* < 0.001). Multiple linear regression analysis was used to examine the association between gestational weight gain and parameters such as placental weight, volume, and PFR (Table 2). Specifically, in the underweight, normal, and overweight pre-pregnancy groups, for every 1 kg increase in gestational weight gain, the placental weight increased by 4.93 g (95% CI: 1.04–8.81), 2.52 g (95% CI: 1.04–3.99), and 3.30 g (95% CI: 0.38–6.22), respectively. Additionally, for every 1 kg rise in gestational weight gain, the placental volume increased by 6.79 cm³ (95% CI: 3.43–10.15) in the underweight group and 2.85 cm³ (95% CI: 1.31–4.39) in the normal group. The results from sensitivity analysis supported these findings (Table S1).

**Table 2:** The associations of gestational weight gain with placental weight, placental volume, and PFR.

Dependent variable	Gestational weight gain	Model 1 (95%CI)	Model 2 (95%CI)
	Pre-pregnancy BMI group		
Placenta weight	Wasting	5.26 (1.61, 8.90) **	4.93 (1.04, 8.81) *
	Normal	2.93 (1.48, 4.38) **	2.52 (1.04, 3.99) **
	Overweight	3.82 (1.05, 6.58) **	3.30 (0.38, 6.22) *
	Obese	2.72 (–2.63, 8.07)	1.12 (–4.47, 6.70)
Placenta volume	Wasting	7.36 (4.17, 10.56) **	6.79 (3.43, 10.15) **
	Normal	2.73 (1.23, 4.23) **	2.85 (1.31, 4.39) **
	Overweight	1.53 (–1.50, 4.55)	0.55 (–2.64, 3.73)
	Obese	2.89 (–2.95, 8.74)	2.11 (–4.60, 8.83)
PFR	Wasting	0.02 (–0.09, 0.12)	0.01 (–0.10, 0.12)
	Normal	–0.02 (–0.06, 0.02)	–0.02 (–0.06, 0.02)
	Overweight	–0.01 (–0.07, 0.06)	–0.01 (–0.08, 0.06)
	Obese	–0.13 (–0.25, − 0.01) *	–0.12 (–0.25, 0.02)

Model 1: univariate analysis; Model 2: multivariate analysis adjusted for maternal education level, residence, age, gestational hypertension, gestational diabetes, gestational age, number of pregnancies, number of deliveries, infant sex and mid-trimester lipid levels; *p < 0.05, **p < 0.001.

Abbreviations: BMI, body mass index. PFR, placental-to-birth weight ratio.

### Associations of mid-trimester lipid levels with placental weight, placental volume, and PFR

Multiple linear regression was used to investigate the associations between mid-trimester lipid levels and parameters like placental weight, placental volume, and PFR (Table 3). In the adjusted model (model 2), a statistically significant association persisted between placental weight and maternal mid-trimester TG and HDL-C levels. Specifically, placental weight increased as TG levels rose (β = 9.81, 95% CI: 3.28–16.34) and decreased with rising HDL-C levels (β=–46.30, 95% CI: − 69.49 to − 23.11). Before adjusting for confounders (model 1), placental volume was linked to both TG and HDL-C levels. However, after adjustments, only the association with mid-trimester TG levels remained, where placental volume escalated with increasing TG levels (β = 14.54, 95% CI: 7.69–21.39). For PFR in the adjusted model (model 2), it was exclusively associated with mid-trimester HDL-C levels, showing a decrease with increased HDL-C levels (β=–0.89, 95% CI: − 1.50 to − 0.27). Notably, the relationship with LDL-C levels was no longer significant. Sensitivity analysis indicated these findings were stable (Table S2).

**Table 3:** The associations of lipid levels and placental weight, placental volume, and PFR in mid-trimester

Dependent variable	Mid-trimester maternal lipid profile	Model 1 (95%CI)	Model 2 (95%CI)
Placenta weight	TC	5.03 (–0.65, 10.71)	4.93 (–0.66, 10.52)
	TG	11.46 (5.12, 17.80)**	9.81 (3.28, 16.34) **
	HDL-C	–50.29 (–73.84, − 26.73)**	–46.30 (–69.49, − 23.11) **
	LDL-C	4.92 (–1.48, 11.32)	5.47 (–0.83, 11.77)
Placenta volume	TC	–0.29 (–6.21, 5.63)	0.70 (–5.19, 6.59)
	TG	17.79 (11.21, 24.36)**	14.54 (7.69, 21.39) **
	HDL-C	–29.18 (–53.85, − 4.50)*	–21.24 (–45.77, 3.30)
	LDL-C	–2.78 (–9.46, 3.90)	–0.95 (–7.59, 5.70)
PFR	TC	0.14 (–0.01, 0.29)	0.11 (–0.03, 0.26)
	TG	0.06 (–0.10, 0.23)	0.13 (–0.04, 0.31)
	HDL-C	–0.84 (–1.45, − 0.23)**	–0.89 (–1.50, − 0.27)**
	LDL-C	0.18 (0.02, 0.35)*	0.15 (–0.02, 0.32)

Model 1: univariate analysis; Model 2: multivariate analysis adjusted for maternal education, residence, age, pre-pregnancy BMI, gestational hypertension, gestational diabetes, gestational age, number of pregnancy, number of delivery, infant sex and gestational weight gain; *p < 0.05, **p < 0.001.

Abbrevations: TC, cholesterol. TG, triglyceride. HDL-C, high-density lipoprotein. LDL-C, low-density lipoprotein. PFR, placental-to-birth weight ratio.

## Discussion

In our study involving 1,358 mother-infant pairs, we meticulously measured placental weight, placental volume, and newborn birth weight, subsequently calculating the PFR. Our findings indicate that both placental weight and volume are correlated with gestational weight gain and mid-trimester lipid levels, while PFR is linked to mid-trimester lipid levels. For those pregnant women who were underweight prior to pregnancy, the influence of gestational weight gain on placental weight was more pronounced than for those with a normal or overweight pre-pregnancy BMI. Similarly, for women underweight before pregnancy, the impact of gestational weight gain on placental volume surpassed that of women with a normal pre-pregnancy BMI. Moreover, both placental weight and volume showed positive correlations with mid-trimester TG levels. Conversely, both placental weight and PFR demonstrated negative associations with mid-trimester HDL-C levels.

The fetal intrauterine development and growth are largely dictated by the nutrients the mother transmits through the placenta. The nutrient transport relies both on placental size and its efficiency. In this study, placental weight and volume represented placental size, while PFR was utilized as a measure of its efficiency [26]. Given the influence of pre-pregnancy BMI on gestational weight gain, we conducted a subgroup analysis based on this parameter. Consistent with prior research [27, 28], maternal gestational weight gain influenced placental weight and volume. However, in our subgroup analysis, there was no correlation between placental weight and maternal gestational weight gain in the obese category, and between placental volume and maternal gestational weight gain in the overweight-obese category. Interestingly, underweight women prior to pregnancy exhibited the most pronounced effect of gestational weight gain on placental weight and volume. This might stem from the leaner body composition and metabolism of such women, making it challenging for them to accumulate fat. As a result, post-pregnancy, the fats consumed are more readily absorbed by the placenta. Additionally, underweight pregnant women, being more attentive to nutritional supplementation post-pregnancy compared to overweight or obese women, tend to gain more weight during this period. The underweight category often has a paucity of essential nutrients, leading to smaller placentas [29]. To sustain regular fetal growth, the placenta augments its capillary tissue and nutrient transport capacity. This suggests that the development and angiogenesis of placenta become increasingly reliant on gestational weight gain [30].

In comparing placental weight and volume across different age brackets, it emerged that older women (> 35 years old) had placentas with the lowest weight but the largest volume. Some researchers found the association of advanced maternal age with deficient placental α-klotho expression, leading to placental weight reductions and late-gestation malformations [31]. This malformation, inducing placental shape irregularities, results in inaccuracies when determining placental volume. Despite having the lightest weight, why pregnant women aged more than 35 years old had the largest placental volume remains an area requiring further research. Previous studies posited that a lighter placenta supports more fetal mass per gram than a heavier one, implying that higher placental efficiency actually denotes a slower placental growth rate. Our research revealed that gestational weight gain did not impact PFR, irrespective of pre-pregnancy BMI. Though diet has been shown to influence placental efficiency, our study did not collect data on pregnant women’s dietary habits.

Previous research has demonstrated that placentas with a lighter mass support approximately 20% more fetal mass per gram compared to heavier placentas [32]. This suggests that a greater placental efficiency is indicative of a slower placental growth rate. Placental efficiency is measured using two primary indicators: placental weight and neonatal weight. In our investigation, maternal weight gain during pregnancy did not influence the PFR, irrespective of the pre-pregnancy BMI. Research conducted by Alwasel et al. and Roseboom et al. [33, 34] identified diet as a factor affecting placental efficiency; however, our research did not gather data on the dietary habits of expectant mothers. The relationship between gestational weight gain and PFR, and any unidentified potential influences between them, warrants exploration in subsequent studies. Hence, it is imperative to develop gestational weight intervention strategies tailored to diverse populations. Appropriate gestational weight gain can ensure optimal placental function, decrease the likelihood of unfavorable pregnancy outcomes, and mitigate adverse long-term impacts on neonates.

Our results revealed that elevated mid-trimester TG levels corresponded with increased placental weight and volume. Additionally, decreased HDL-C levels were associated with heightened placental weight and PFR. This suggests a robust association between placental size, efficiency, and maternal lipid levels. Prior studies have documented a positive correlation between neonatal birth weight and placental weight [6, 35, 36]. Moreover, placental volume is a significant predictor of adverse pregnancy outcomes, such as fetal distress and low Apgar scores [8, 37, 38]. Consequently, grasping changes in placental size and efficiency is paramount for the neonate’s future growth and development. Maternal lipid levels strongly predict fetal growth, implying that placental lipid transfer contributes to fetal overgrowth. Neonatal obesity correlates with the maternal fat delivered to the placenta, including high TG and low HDL-C [39]. Though TGs cannot traverse the placenta, placental lipoprotein lipase decomposes them into free fatty acids. These fatty acids, which fuel both placental and fetal development, are then transmitted to the fetus [40]. This relationship underscores the association between maternal blood TG levels and placental size. Excessive free fatty acids, however, contribute to insulin resistance. Increased fetal insulin resistance, combined with accumulated body fat and glucose, leads to elevated birth weight [41]. A cohort study from Beijing substantiated maternal TG levels as a significant predictor of fetal body fat [42]. In our sample, over half of the pregnant participants exhibited abnormal TG levels. It is worth noting that excessive lipid accumulation might compromise placental integrity and function [16, 17], leading to adverse pregnancy outcomes.

Given the anti-atherogenic and anti-inflammatory characteristics of HDL-C, it’s plausible that diminished HDL-C during gestation could amplify inflammation [43]. This inflammatory response might then mediate changes in placental density and vascular functionality, which could account for the weight increase linked to low HDL-C levels. Mudd et al. reported a correlation between higher mid-trimester HDL-C and reduced birth weight [44]. This implies that, in environments with low HDL-C, the placenta may be less efficient, supporting lower fetal weight. In our study, HDL-C exhibited notable regression coefficient values, possibly due to data sparsity. To enhance the precision, we aim to expand the sample size in subsequent research. Factors contributing to dyslipidemia during pregnancy are multifaceted, encompassing age, pre-pregnancy BMI, gestational weight gain, diet, exercise, and beyond. Numerous studies have spotlighted higher serum TG concentrations and reduced HDL-C levels in obese pregnant women compared to those with lower pre-pregnancy weights [45, 46]. Such obese individuals often exhibit diminished metabolic adaptations, potentially exacerbating dyslipidemia. Moreover, excessive weight gain is acknowledged as a risk factor for dyslipidemia [47]. To elucidate distinct effects of mid-pregnancy TG and HDL on placental morphology and efficiency, our analysis controlled for pre-pregnancy BMI and gestational weight gain.

At present, our study has not established a significant linear relation between TC and LDL-C levels with placental size and efficiency. Nonetheless, the non-linear association between these variables will be a focal point in subsequent research. Elevated serum TC and LDL-C levels in pregnant women have been linked to altered placental vasculature [48]. With a 62.8% hyperlipidemia prevalence in our study population (Table S3), there is an urgent need for enhanced health surveillance during pregnancy. The ramifications of gestational dyslipidemia extend beyond adverse birth outcomes, impacting the offspring’s future cardiovascular health, and risks of obesity or hypertension [49].

Few studies have explored the relationship between lipid levels and placental development during pregnancy. This gap in the literature can be attributed to several factors: the absence of specific domestic diagnostic criteria for dyslipidemia tailored to pregnant individuals and the infrequent assessment of lipid levels during pregnancy. Yet, the significance of examining lipid levels in pregnancy is escalating, given their potential influence—particularly cholesterol, triglycerides, and lipoproteins—on placental functionality and efficiency. The precise mechanisms through which lipid levels influence the PFR in mid-pregnancy remain to be elucidated. A potential mechanism might involve anomalies in the placental vascular structure. Researchers have noted an elevated expression of VEGF and CD31 in the placentas associated with pregnancies complicated by GDM. This elevation was also found to be independently linked with maternal BMI and pregnancy weight gain [50]. A hyperglycemic state can lead to impaired hypoxia. This hypoxic state, in turn, boosts VEGF expression, promoting placental neovascularization [51]. As a result, women with GDM often exhibit larger and heavier placentas. Furthermore, a study conducted by Dubova et al. [52] revealed that the expression of VEGF and VEGFR-2 in obese women’s placentas surpasses that of their normal-weight counterparts. Given that obesity is a significant risk factor for GDM, disruptions in lipid metabolism—like elevated triglyceride levels and decreased HDL-C—coupled with excessive pregnancy weight gain, may enhance neoangiogenesis and the expression of inflammatory markers. Such changes can lead to alterations in both the structure and function of the placenta.

The present study prospectively collected comprehensive data on pregnant women from early pregnancy through to delivery, thus offering a more robust causal foundation for the impact of gestational weight gain and mid-trimester lipid levels on placental development. In addition, we analyzed variables including placental weight, placental volume, and PFR to comprehensively assess the influences of gestational weight gain and mid-trimester lipid levels on placental development. This approach overcame the constraints of prior studies that only assessed a single placental indicator. However, our study is not without limitations. Notably, we did not capture data on maternal dietary habits during pregnancy. Consequently, potential modifications in lipid levels and placental efficiency attributable to dietary conditions were neither identified nor adjusted for. While we averaged placental dimensions from multiple measurements to mitigate measurement bias, inaccuracies in assessing placental morphology could attenuate the associations observed with our independent variables. Furthermore, as our findings are drawn from a single hospital, our sample is geographically limited and may not fully represent the broader population, potentially introducing bias. This confines the generalizability of our results to other regions. Future research could undertake a multicenter study, gather information on the dietary habits of expectant mothers, and enhance the precision of placental size measurements to more confidently validate the findings of our current investigation.

## Conclusions

In the present study, we found preliminary evidence suggesting that gestational weight gain and mid-trimester lipid levels influence placental weight, volume, and PFR. The impact of gestational weight gain on placental weight and volume appears to be modulated by the mother’s pre-pregnancy BMI. Furthermore, mid-trimester TG and HDL-C levels exhibit a strong correlation with placental metrics. To mitigate pregnancy complications and prevent adverse outcomes for both mother and newborn, it is clinically vital to initiate early BMI interventions, and to closely monitor weight and lipid levels throughout pregnancy. As such, it is imperative to ensure moderate weight gain during pregnancy, maintain optimal blood lipid levels, and conduct regular lipid screenings. The evidence from this study offers a robust scientific foundation for weight and lipid management strategies in expectant mothers.

### Electronic supplementary material

Below is the link to the electronic supplementary material.

Supplementary Material 1

## Data Availability

The datasets used and/or analyzed during the current study are available from the corresponding authors on reasonable request.

## Abbreviations

PFR: Placental-to-birth weight ratio
TG: Triglyceride
HDL-C: High-density lipoprotein
LDL-C: Low-density lipoprotein
TC: Cholesterol
