# Supplementary material for: The associations of gestational weight gain and midpregnancy lipid levels with placental size and placental-to-birth weight ratio: findings from a chinese birth cohort study
Source: BMC Pregnancy Childbirth. 2023 Oct 11;23:725. doi: 10.1186/s12884-023-05991-x (PMC10568921; doi:10.1186/s12884-023-05991-x)
Supplement: Supplementary file 1 — Supplementary Material 1 [file 12884_2023_5991_MOESM1_ESM.docx]

| **Table of Contents** | |
| --- | --- |
| **Table S1** | Sensitivity analysis of regression models of gestational weight gain and placental weight, placental volume, and PFR. |
| **Table S2** | Sensitivity analysis of regression models of mid-trimester lipid levels and placental weight, placental volume, and PFR. |
| **Table S3** | Mid-trimester dyslipidemia table. |

**Table S1** Sensitivity analysis of regression models of gestational weight gain and placental weight, placental volume, and PFR

| **Dependent variable** | **Pre-pregnancy BMI** | **Model 1 (95%CI)** | **Model 2 (95%CI)** | **Model 3 (95%CI)** | **Model 4 (95%CI)** |
| --- | --- | --- | --- | --- | --- |
| Placenta weight | Wasting | 4.92 (1.12,8.72) * | 4.96 (1.13, 8.79) * | 4.96 (1.12, 8.81) * | 5.00 (1.12,8.89) * |
|  | Normal | 2.73 (1.27,4.19) ** | 2.85 (1.37, 4.34) ** | 2.83 (1.35, 4.31) ** | 2.54 (1.06,4.01) ** |
|  | Overweight | 3.98 (1.13,6.83) ** | 3.58 (0.70, 6.47) * | 3.53 (0.61, 6.44) * | 3.57 (0.71,6.43) * |
|  | Obese | 2.61 (-2.84,8.06) | 2.15 (-3.27, 7.57) | 3.09 (-2.42, 8.60) | 2.25 (-3.30,7.79) |
| Placenta volume | Wasting | 6.83 (3.50,10.16) ** | 6.78 (3.46, 10.11) ** | 6.82 (3.50, 10.15) ** | 7.03 (3.65,10.42) ** |
|  | Normal | 2.72 (1.20,4.24) ** | 3.04 (1.51, 4.57) ** | 3.03 (1.49, 4.57) ** | 2.79 (1.25,4.32) ** |
|  | Overweight | 1.74 (-1.38,4.86) | 1.06 (-2.05, 4.17) | 0.92 (-2.22, 4.06) | 0.73 (-2.42,3.89) |
|  | Obese | 3.10 (-2.86,9.06) | 3.03 (-3.09, 9.14) | 3.13 (-3.18, 9.44) | 3.01 (-3.56,9.57) |
| PFR | Wasting | 0.01 (-0.10,0.12) | 0.01 (-0.11, 0.12) | 0.01 (-0.11, 0.11) | 0.01 (-0.11,0.11) |
|  | Normal | -0.02 (-0.06,0.02) | -0.03 (-0.07, 0.02) | -0.03 (-0.07, 0.01) | -0.02 (-0.06,0.02) |
|  | Overweight | -0.01 (-0.07,-0.06) | -0.01 (-0.08, 0.06) | -0.01 (-0.08, 0.05) | -0.01 (-0.08,0.06) |
|  | Obese | -0.12 (-0.25,0.01) | -0.14 (-0.26, 0.01) | -0.12 (-0.25, 0.01) | -0.10 (-0.23,0.04) |

Model 1: multivariate analysis adjusted for maternal education, place of residence and age;

Model 2: multivariate analysis adjusted for maternal education, place of residence, age, gestational hypertension and gestational diabetes;

Model 3: multivariate analysis adjusted for maternal education, place of residence, age, gestational hypertension, gestational diabetes, number of pregnancies and number of deliveries;

Model 4: multivariate analysis adjusted for maternal education, place of residence, age, gestational hypertension, gestational diabetes, number of pregnancies, number of deliveries, week of delivery, and sex of the newborn;

*p<0.05, **p<0.01.

**Table S2** Sensitivity analysis of regression models of mid-trimester lipid levels and placental weight, placental volume, and PFR

| **Dependent variable** | **Mid-pregnancy blood lipid indicators** | **Model 1 (95%CI)** | **Model 2 (95%CI)** | **Model 3 (95%CI)** | **Model 4 (95%CI)** |
| --- | --- | --- | --- | --- | --- |
| Placenta weight | TC | 5.96 (0.26,11.66) * | 5.94 (0.23, 11.65) * | 5.81 (0.08, 11.53) * | 5.28 (-0.33, 10.89) |
|  | TG | 9.37 (2.80,15.97) ** | 9.81 (3.13, 16.49) ** | 9.91 (3.23, 16.60) ** | 9.26 (2.68, 15.84) ** |
|  | HDL-C | -47.20 (-70.84,-23.55) ** | -47.57 (-71.28, -23.86) ** | -46.99 (-70.77, -23.22) ** | -44.89 (-68.18, -21.60) ** |
|  | LDL-C | 6.16 (-0.27,12.60) | 6.13 (-0.32, 12.57) | 6.01 (-0.45, 12.47) | 5.47 (-0.87, 11.81) |
| Placenta volume | TC | 1.34 (-4.61,7.30) | 1.46 (-4.49, 7.42) | 1.39 (-4.58, 7.36) | 0.99 (-4.93, 6.91) |
|  | TG | 14.96 (8.12,21.80) ** | 14.40 (7.47, 21.34) ** | 14.47 (7.52, 21.41) ** | 13.96 (7.04, 20.87) ** |
|  | HDL-C | -23.06 (-47.82,1.69) | -21.70 (-46.50, 3.10) | -21.59 (-46.46, 3.28) | -20.50 (-45.15, 4.15) |
|  | LDL-C | -0.80 (-7.52,5.91) | -0.54 (-7.26, 6.19) | -0.62 (-7.35, 6.11) | -0.95 (-7.63, 5.73) |
| PFR | TC | 0.12 (-0.03,0.26) | 0.12 (-0.03, 0.26) | 0.10 (-0.05, 0.25) | 0.11 (-0.04, 0.25) |
|  | TG | 0.12 (-0.05,0.79) | 0.12 (-0.05, 0.30) | 0.13 (-0.04, 0.31) | 0.15 (-0.02, 0.33) |
|  | HDL-C | -0.95 (-1.56,-0.34) ** | -0.95 (-1.57, -0.34) ** | -0.88 (-1.50, -0.27) ** | -0.88 (-1.49, -0.26) ** |
|  | LDL-C | 0.16 (-0.01,0.32) | 0.16 (-0.01, 0.32) | 0.14 (-0.02, 0.31) | 0.15 (-0.02, 0.31) |

Model 1: multivariate analysis adjusted for maternal education, place of residence, age and pre-pregnancy BMI;

Model 2: multivariate analysis adjusted for maternal education, place of residence, age, pre-pregnancy BMI, gestational hypertension and gestational diabetes;

Model 3: multivariate analysis adjusted for maternal education, place of residence, age, pre-pregnancy BMI, gestational hypertension, gestational diabetes, number of pregnancies and number of deliveries;

Model 4: multivariate analysis adjusted for maternal education, place of residence, age, pre-pregnancy BMI, gestational hypertension, gestational diabetes, number of pregnancies, number of deliveries, week of delivery, and sex of the newborn;

*p<0.05, **p<0.01.

**Table S3** Mid-trimester dyslipidemia table.

|  | **Exception Criteria** | **Number of people** | **Percentage** |
| --- | --- | --- | --- |
| TC | ≥6.21 | 401 | 29.5% |
| TG | ≥2.26 | 697 | 51.3% |
| HDL-C | ≤1.04 | 2 | 0.1% |
| LDL-C | ≥4.14 | 171 | 12.6% |
| Dyslipidemia |  | 853 | 62.8% |
